# P-2017. Patient Reported Outcomes of Nirmatrelvir/Ritonavir Treatment for High-risk, Nonhospitalized Adults with Symptomatic COVID-19

**DOI:** 10.1093/ofid/ofae631.2174

**Published:** 2025-01-29

**Authors:** Ashley S Cha-Silva, Henriette Coetzer, Kelly Gebo, Jinma Ren, Amie Scott, Joseph C Cappelleri, Wajeeha Ansari, Heidi Leister-Tebbe

**Affiliations:** Pfizer, New York, New York; Blue Health Intelligence, Chicago, Illinois; Johns Hopkins, Baltimore, MD; Pfizer Inc., Collegeville, Pennsylvania; Pfizer, Inc, New York, New York; Pfizer Inc., Collegeville, Pennsylvania; Pfizer Inc., Collegeville, Pennsylvania; Pfizer Inc, Collegeville, Pennsylvania

## Abstract

**Background:**

Nirmatrelvir/ritonavir (NMV/r) is an oral antiviral treatment for coronavirus disease 2019 (COVID-19). We describe patient-reported outcomes (PROs) with NMV/r in nonhospitalized adults with COVID-19 at high risk of severe disease.

Percentage of participants in each treatment group in the mITT2 population reporting return to usual health on each study day through Day 28 on the Global Impressions Questionnaire.

**Figure:**
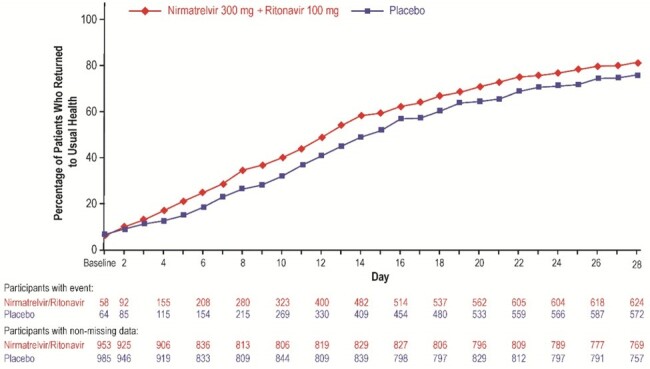

n=number of patients with event; mITT2=modified intent-to-treat 2; N=number of patients with nonmissing data. CI=confidence interval; HR=hazard ratio

**Methods:**

In a phase 2/3 double-blind study, high-risk adults with confirmed COVID-19 and ≤ 5 days of symptoms were randomized 1:1 to receive NMV/r or placebo twice daily for 5 days. All PRO analyses included participants randomly assigned to a study intervention who received ≥ 1 dose of the study intervention and had ≥ 1 postbaseline visit through Day 28 (modified intent-to-treat 2 [mITT2] population). Patients recorded responses to three Global Impression Questions (GIQ) daily through Day 28 to assess return to usual health, return to usual activities, and overall severity of COVID-19 related symptoms. COVID-19–specific Work Productivity and Activity Impairment Questionnaire (WPAI-COVID-19), and the EuroQol Quality of Life 5-Dimension 5-Level Scale (EQ-5D-5L) were completed at specified visits through Week 24.

Percentage of participants in each treatment group in the mITT2 population reporting return to usual activities on each study day through Day 28 on the Global Impressions Questionnaire.

**Figure:**
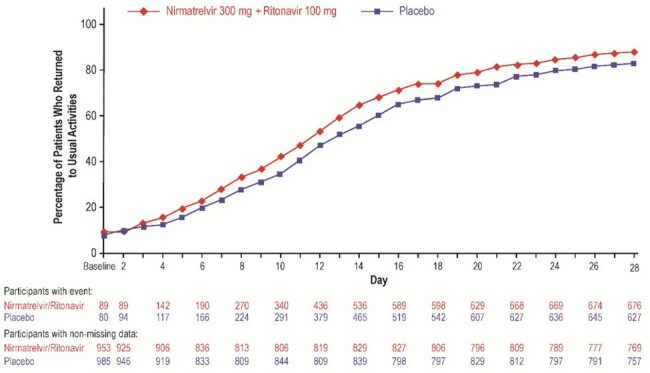

n=number of patients with event; N=number of patients with nonmissing data. CI=confidence interval; HR=hazard ratio; mITT2=modified intent-to-treat 2

**Results:**

Compared with placebo, patients receiving NMV/r had a significant 3-day reduction in median time to return to usual health (hazard ratio [HR], 1.3; 95% confidence interval [CI], 1.2–1.4), 1-day reduction in median time to return to usual activities (HR, 1.2; 95% CI, 1.1–1.4), and significantly shorter time to sustained resolution of any overall symptoms (HR, 1.2; 95% CI, 1.1–1.3) and sustained alleviation of any overall symptoms (HR, 1.2; 95% CI, 1.1–1.3) based on the GIQ. There were no significant differences between treatment groups on the WPAI or EQ-5D-5L.

**Conclusion:**

High risk patients receiving NMV/r for COVID-19 reported a reduced duration and severity of COVID-19 symptoms based on their global impression of overall symptom burden and quicker return to usual heath and usual activities compared with those receiving placebo. The main limitation of this analysis was the lack of sufficient ePRO data collection of WPAI and EQ-5D-5L questionnaires. As such, no meaningful conclusions could be drawn regarding the impact of NMV/r on work productivity or activity impairment of HRQoL.

**Disclosures:**

Ashley S. Cha-Silva, PharmD, MS, Pfizer Inc.: Employee|Pfizer Inc.: Stocks/Bonds (Public Company) Jinma Ren, PhD, Pfizer Inc.: Employee|Pfizer Inc.: Stocks/Bonds (Public Company) Amie Scott, MPH, Pfizer: Employee|Pfizer: Stocks/Bonds (Public Company) Joseph C. Cappelleri, PhD, Pfizer Inc.: Employee|Pfizer Inc.: Stocks/Bonds (Public Company) Wajeeha Ansari, MPH, Pfizer: Employee|Pfizer: Stocks/Bonds (Public Company) Heidi Leister-Tebbe, BSN, Pfizer: Employee|Pfizer: Stocks/Bonds (Public Company)

